# Endoscopic Resection of Gastric Submucosal Masses by a Dental Floss Traction Method

**DOI:** 10.1155/2019/1083053

**Published:** 2019-05-02

**Authors:** Chunyan Zeng, Yin Zhu, Xu Shu, Nonghua Lv, Qiang Cai, Youxiang Chen

**Affiliations:** ^1^Department of Gastroenterology, The First Affiliated Hospital of Nanchang University, Nanchang, China; ^2^Division of Digestive Diseases, Emory University School of Medicine, Atlanta, GA, USA

## Abstract

**Background and Aims:**

ESE (endoscopic submucosal excavation) is widely used for the treatment of digestive diseases. The dental floss traction (DFT) method has been successfully used to facilitate ESE to resect mucosal lesions such as early gastric cancer. DFT has not been used in ESE to remove submucosal masses. This study aimed to examine the efficacy of DFT-assisted ESE (DFT- ESE) for the removal of submucous masses.

**Methods:**

From March 2017 to May 2017, a total of 12 patients with gastric submucosal masses at the First Affiliated Hospital of Nanchang University, Jiangxi, China, were enrolled. The tumor characteristics, en bloc resection rates, complications, and outcomes on follow-up were evaluated for all patients.

**Results:**

The 12 submucosal tumors were completely removed by DFT- ESE. Nine were gastrointestinal stromal tumors. Two were Schwannoma, located in the greater curvature of the gastric corpus. One was gastric ectopic pancreas. All the resected tumors were removed completely with intact tumor capsules. There was no more bleeding or perforation after the endoscopic closure of the perforation or the wound after the DFT-ESE, and no recurrences were identified at the time of follow-up.

**Conclusions:**

The DFT method efficiently and safely facilitated the ESE procedure during the resection of gastric submucosal tumors. This study was registered with Chinese Clinical Trial Registry under Registration number ChiCTR-OOC-15005833).

## 1. Introduction

Endoscopic submucosal excavation (ESE) has been widely used for resection of the early gastric cancer, gastric submucosal masses, and colonic laterally spreading tumor (LST) [1]. The procedure can be very difficult to perform in some situations, such as when the lesions are located in the gastric fundus or in the greater curvature of the anterior gastric corpus wall or when the lesions cannot be separated from the serous layer (extraluminal growth). Furthermore, some parts of the lesions can fall into the abdominal cavity. Dental floss traction (DFT) has been successfully used to facilitate endoscopic submucosal excavation (ESE)(DFT-ESE) to remove mucosal lesions, such as early gastric cancer [2–6]. However, to our knowledge, DFT-ESE has not been used in resection of submucosal masses. This study aimed to identify the efficacy of DFT-ESE for the removal of submucosal masses.

## 2. Patients and Methods

From March 2017 to May 2017, twelve patients with gastric submucosal masses located in the gastric fundus or at the greater curvature of anterior gastric corpus wall were enrolled in the study, since lesions located in those locations are difficult to remove by ESE without traction.

The gastric masses were examined by endoscopic ultrasound and computed tomography before ESE; all masses were confirmed to be localized in the submucosal or muscular layer without distant metastasis. The mass characteristics, en bloc resection rate, and complications were reviewed. Informed consent was obtained from each patient.

**Figure 1:**
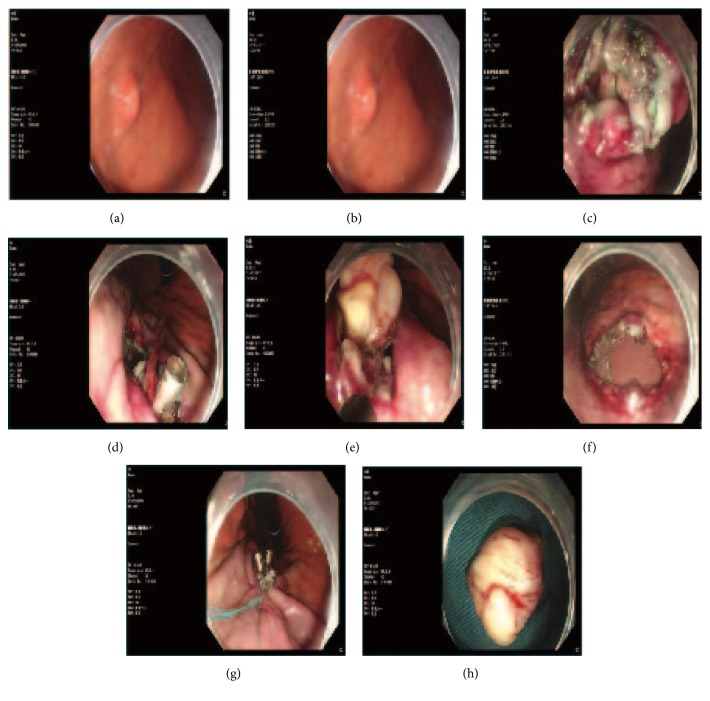
ESD with dental floss clip traction. (a) A bump was seen in the anterior gastric corpus wall (endoscopic ultrasound showed it originated from the muscular layer and grows extraluminally, 2.0cm in diameter), labeled with Hook Knife. (b) The mass was showed after Hook knife precutting the mucous layer. (c) Strip off the mass. (d) The mass was pulled by the dental floss clip. (e) The lesion clearly exposed with the dental floss traction. The lesion was easier to remove en bloc with hook knife. (f) The post-ESD wound has no defect left. A large perforation was seen. (g) The wound was large and closed with nylon loop pouch-suture through a single channel endoscope. (h) The tumor.

**Figure 2:**
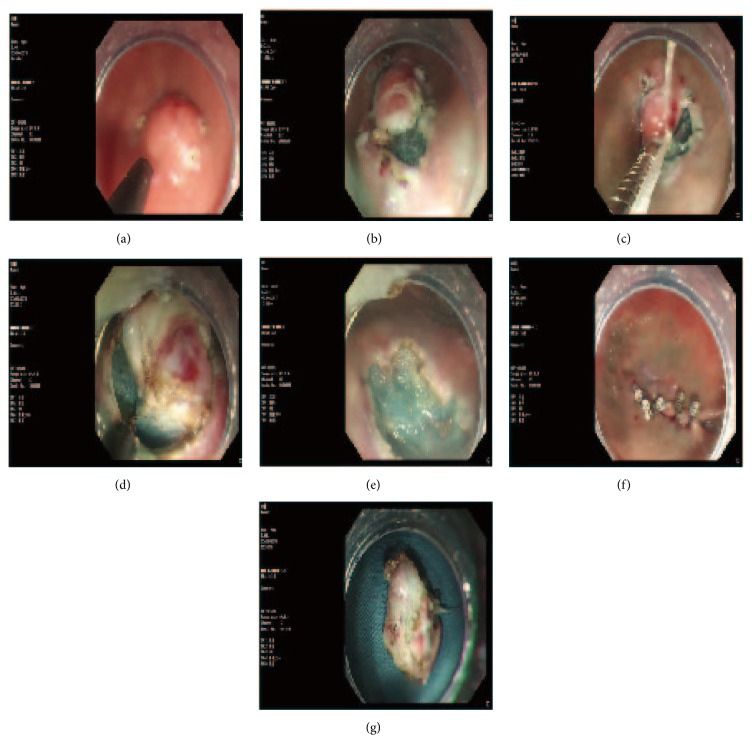
ESD with dental floss clip traction. (a) A bump was seen in the gastric fundus (endoscopic ultrasound showed it originated from the muscular layer, 1.5cm in diameter), labeled with Hook Knife. (b) Hook knife cut off most part of the mass along the labeled margin, while the endoscopic transparent cap could not enter into the gap between the lesion and normal tissue, which led to difficulty of the resection. (c) The clip fixed the dental floss right in front of the endoscopic vision. (d) After the traction, the lump was clearly defined by the normal tissue, and the HK knife was easy to peel off the lump. (e) The post-ESD wound has no defect left. (f) The wound was closed with titanium clips. (g) The mass.

The DFT-ESE procedure is depicted in the images presented in Figures 1 and 2. A detailed description is as follows.

First, the mass was labeled and injected in multipoint with lifting solution (containing 250 ml glycerin fructose, 3 mg adrenalin, and 5 mg methylene blue) by injection needle through an endoscope (GIF-Q260J, Olympus) channel. The mass body was usually identified after the mucosa was dissected along the label margin. Second, dental floss was knotted to the titanium clip (HX-610-135; Olympus, Aomori, Japan), which was then delivered to the lesion through the biopsy channel of the endoscope. The titanium clip was clamped at the side of the mass, and the lifting position of the mass was kept in front of the endoscopic view while pulling. Lastly, we used varying levels of strength to pull the dental floss according to the exposure extent of the mass. In this way, the hook knife (KD-620LR/Q/U; Olympus) could easily enter into the gap between the mass and normal tissue and therefore, the mass could be easily resected en bloc. During ESE, hemostasis was achieved with HybridKnifes (ERBE-VIO200D, Tuebingen, Germany) or Coagrasper (FD-410LR/FD-411QR, Olympus). The method used for closing the wound or perforation depended on its size. Small wounds or perforations were directly closed with titanium clips, whereas for large ones, we used endoscopic nylon loop and a titanium clips pouch suture technique to close them, which we have reported in our previous study [7, 8].

After the operation, all patients who underwent full-thickness resection or had perforation during the operation were fasted for 24 hours and received antibiotics for 24-48 hours and proton pump inhibitor (PPI) therapy for 4-6 weeks. For the patients without perforation, they fasted for 24 hours after the procedure and were given PPI for 4-6 weeks without antibiotics.

## 3. Results

From March 2017 to May 2017, our group had completed 12 cases with gastric submucosal mass by DFT-ESE.The details are shown in Table 1. Twelve patients were enrolled in the group (male:female=5:7), with ages ranging from 38 to 72 years old (average age: 53). Five of them underwent gastric fundus full-thickness resection and five underwent with gastric body full-thickness resection. All of the patients received en bloc resection with one attempt by DFT-ESE. Complications, such as bleeding or infection, did not occur. The diameters of the lesions ranged from 1.0 to 2.5 cm (average: 1.5cm).

## 4. Discussion

Gastric submucosal masses include gastric stromal tumor, leiomyoma, heterotopic pancreas, neuroendocrine tumor, and lipoma. Some of the lesions, such as gastric stromal tumors, have malignancy potential. At present, endoscopic submucosal dissection (ESD), ESE, and endoscopic full-thickness resection (EFR) are used to remove those tumors [9–12], but some lesions, due to the location, may not be easily removed by ESE, especially by inexperienced hands. Possible reasons include the following: the endoscope cannot reach the lesion, for instance, lesions in the gastric fundus, in some areas of the greater curvature, or in anterior wall of the gastric body; some lesions after resection may fall into the abdominal cavity; some lesions grow outward from the lumen. All of those situations often cause failure of ESE. Dental floss traction was first used to facilitate ESE in resection of mucosal lesions, such as early gastric cancer. It is not widely applied because it may cause damage to the lesion by pulling too hard. Currently, it is reported that DFT-ESE could reduce the risk of perforation and procedure time [2–4, 6, 13]. Our study reported the facilitating effect of DFT on ESE removal of gastric submucosal masses.

The key step that ensures DFT-ESE successful is that the transparent cap attached at the tip of the endoscope can get close to the gap between the lesion and the normal tissue after traction. For lesions located in the gastric fundus or in the greater curvature of anterior gastric corpus wall, it is difficult to reach to lesion with the endoscope during conventional ESE procedure. For lesions arising from the serous layer, the transparent cap does not maintain a good view by separating the lesion from the normal tissue because the scope and cap cannot reach the gap between the lesion and the normal tissue, resulting in difficulty and even failure to remove the lesions.

DFT can assist in exposing the gap between the lesion and the normal tissue by lifting the lesion, thereby making resection possible, even if the endoscope cannot reach the lesion or the transparent cap cannot enter into the gap between the lesion and the normal tissue, even if, during the process of gastric full-thickness resection of a lesion, DFT can maintain a good view and prevent the lesion from falling into the abdominal cavity. Since DFT makes ESE easier, it improves the success rate of ESE and reduces the complications such as bleeding and perforation which happen during the operation.

We recommend using DFT-ESE for resection of gastric submucosal masses located in some anatomic areas, such as the gastric fundus; the greater curvature of anterior gastric corpus to increases the success rate of resection.

## Figures and Tables

**Table 1:** Clinical data of the patients.

case	Disease	Gender	Age	Location of the disease	size (cm)	the main layer tumor dominated	procedure time (min)	perforation	horizontal margin
1	Schwannoma	male	60	the greater curvature of gastric corpus	1.0	Muscular layer	27	Yes	Negative
2	Schwannoma	female	54	the greater curvature of gastric corpus	2.0	Muscular layer	32	Yes	Negative
3	ectopic pancreas	female	42	Gastric antrum	1.0	Submucosa	19	No	Negative
4	stromal tumor	male	53	Gastric fundus	1.0	Muscular layer	58	Yes	Negative
5	stromal tumor	female	54	Gastric fundus	1.0	Muscular layer	30	Yes	Negative
6	stromal tumor	male	48	Gastric fundus	1.5	Muscular layer	24	Yes	Negative
7	stromal tumor	female	72	Anterior gastric corpus wall	2.0	Muscular layer	30	Yes	Negative
8	stromal tumor	female	49	the greater curvature of anterior gastric fundus wall	1.3	Muscular layer	67	Yes	Negative
9	stromal tumor	female	63	the greater curvature of anterior gastric corpus wall	1.5	Muscular layer	22	Yes	Negative
10	stromal tumor	female	49	Gastric fundus	1.2	Muscular layer	16	No	Negative
11	stromal tumor	male	63	Gastric fundus	1.6	Muscular layer	34	Yes	Negative
12	stromal tumor	male	38	the greater curvature of gastric corpus	2.5	Muscular layer	32	Yes	Negative

## Data Availability

The clinical data was not public to protect the privacy of patients. But the data could be shared when others asked for a reasonable request by e-mail to me.
